# Editorial: Sodium Glucose Co-Transporter 2 Inhibitors and Kidney Function

**DOI:** 10.3389/fphar.2022.915713

**Published:** 2022-05-03

**Authors:** Daniel H. Van Raalte, Hiddo Lambers Heerspink

**Affiliations:** ^1^ Amsterdam University Medical Center, Amsterdam, Netherlands; ^2^ University Medical Center Groningen, Groningen, Netherlands

**Keywords:** diabetes, diabetic kidney disease, SGLT2 (sodium-glucose cotransporter 2) inhibitor, cardiovascular disease, heart failure, chronic kidney disease

Sodium glucose cotransporter 2 (SGLT2) inhibitors were originally developed for the treatment of type 2 diabetes. SGLT2 inhibitors lower blood glucose levels by inducing glucosuria through inhibition of the SGLT2 transporter in the proximal tubule of the kidney. However, through incompletely understood mechanisms, SGLT2 inhibitors provide cardiovascular benefit, reduce heart failure hospitalizations and have kidney protective properties that are present in people with and without diabetes. Thus, it is evident that these drugs have received a central place in guidelines issued by internal medicine specialists, cardiologists and nephrologist. In this Research Topic in Frontiers, we publish six papers that contribute to the understanding of these agents and position in the current treatment paradigm for cardiovascular and kidney disease.

First, two basic science papers are reported that have explored novel mechanisms of action of SGLT2 inhibition with respect to kidney protection. Zhai et al. studied the role of the SGLT2 inhibitor empagliflozin in pregnant mice that developed pre-eclampsia due to injection of autoantibodies against angiotensin II type 1 receptor. Pre-eclampsia is a common cause of maternal and perinatal morbidity and mortality and also is a risk factor for cardiovascular and kidney disease later in life. Empagliflozin reduced blood pressure and albuminuria in the mice suffering from pre-eclampsia and reduced kidney damage in histological examinations of kidney tissue. Interestingly, empagliflozin activated the energy sensor complex AMPK/SIRT1 and downregulated oxidative stress, pathways previously speculated to be involved in the SGLT2 inhibitor protective effects, in the kidney of these mice. A second rodent study explored the effects of dapagliflozin on the innate immune systems. In diabetes, the hyperglycemic milieu results in an inadequate immune response when exposed to pathogens. The complement system is an important part of the innate immune system and over-activation of the complement system seems involved in the pathogenesis of diabetic kidney disease. Chang et al. demonstrated that dapagliflozin compared to vehicle-treated mice upregulates complement receptor type 1-related protein y (Crry), a key complement regulator which inhibits complement over-activation. This effect was accompanied by suppression of hypoxia factor HIF-1α and provides a novel mechanism for how SGLT2 inhibition may confer kidney protection in diabetes.

Clinicians always have a need for suitable biomarkers that reflect a beneficial response to a drug they prescribe. This is no different for SGLT2 inhibition, and several studies are ongoing to develop biomarkers that predict the degree of long-term kidney protection with these agents. Two papers have addressed this topic in this issue, the first paper focused on a plasma metabolite and in the second paper imaging biomarkers are explored. Firstly, Curovic have investigated whether a novel biomarker called soluble urokinase plasminogen activator receptor (suPAR) could reflect the protective properties of SGLT2 inhibition. Elevated suPAR has been strongly linked in prior research to diabetes-related complications. They investigated this in a group of people with type 1 diabetes and albuminuria in a single-dose study and in people with type 2 diabetes with albuminuria following more prolonged treatment with SGLT2 inhibition. Although SGLT2 inhibition induced favorable kidney effects, no changes in suPAR were observed, indicating that it is not a predictive biomarker for the beneficial effects of SGLT2 inhibition. Secondly, the use of imaging biomarkers which reflect the mechanism of action of SGLT2 inhibitors was described by van der Hoek and Stevens in a state-of-the-art review. They have specifically focused on the role of kidney hypoxia in diabetic kidney disease and potential modulation of this pathological process by SGLT2 inhibition. While they provide an up-to-date overview of kidney MRI imaging, they also discuss the potential use of kidney positron emission tomography (PET). These imaging modalities may help to understand the pathophysiology of kidney disease and to allow for monitoring of therapies.

Finally, two clinical studies complete this Research Topic. Understanding how the benefits of SGLT2 inhibitors as observed in clinical trials translate to clinical practice is important to assess the external validity of clinical trial results. In this respect Lee et al. compared the effects of empagliflozin with linagliptin in 7042 people with type 2 diabetes using data from a large healthcare delivery system in Taiwan. Outcomes assessed were acute kidney injury (AKI), post-AKI dialysis and mortality. After careful matching of patients, they found that empagliflozin compared to linagliptin was associated with a 40% relative risk reduction for AKI and a slower decline of kidney function over time. These data support other real world practice database studies which collectively have demonstrated that findings of clinical trials with SGLT2 inhibitors can be generalized to routine clinical care.

Finally, the last chapter of this Research Topic compared the effects of SGLT2 inhibitors with a novel mineralocorticoid receptor antagonist finerenone. Finenerone is a non-steroidal MRA which has been shown to reduce the risk of kidney failure and heart failure in patients with diabetic kidney disease. The network meta-analysis conducted by Zhao et al. aimed to directly compare the efficacy of SGLT2 inhibitors with finerenone. The study suggests that SGLT2 inhibition compared to finerenone is associated with a lower risk of a kidney endpoint defined as a large sustained decline in kidney function of at least 40% or 57%, kidney failure or death due to kidney failure. The authors conclude that SGLT2 inhibitors are more effective than finerenone in reducing cardio-renal events. However, it is important to emphasize that such conclusions have to be cautiously interpreted in the absence of head-to-head trials. In addition, individual patients may respond differently to SGLT2 inhibitors and finerenone and the challenge for clinicians is to find the right drug for the right patient. For some patients this is a SGLT2 inhibitor, for others finerenone and again for others the combination of the two.

To conclude, the studies published have shed a light on novel aspects of SGLT2 inhibition, and have confirmed mechanisms and outcomes published previously. It is expected that SGLT2 inhibitors will be widely prescribed due to their profound protective effects and clinical benefits. Future studies that 1) further explore their mechanism of action, 2) focus on effective treatment combination of SGLT2 inhibitors with other drugs that lower cardiovascular disease and/or kidney disease and 3) identify biomarkers that may predict SGLT2 response in individuals need to be carried out.

